# The role of patient activation in mediating the effects of health literacy level on quality of life among patients with gastrointestinal cancers

**DOI:** 10.1038/s41598-025-91670-0

**Published:** 2025-03-01

**Authors:** Charis Haering, Svenja Heyne, Anja Mehnert-Theuerkauf, Beate Hornemann, Lara Dreismann, Viktoria Ginger, Tanja Zimmermann

**Affiliations:** 1 National Center for Tumor Diseases (NCT/UCC), University Hospital Carl Gustav Carus, Technical University Dresden, Dresden, Germany; 2 Department of Medical Psychology and Medical Sociology, University Medical Center Leipzig, Leipzig, Germany; 3 Comprehensive Cancer Center Central Germany (CCCG), Leipzig, Germany; 4 Department of Psychosomatic Medicine and Psychotherapy, Hannover Medical School, Hannover, Germany

**Keywords:** Gastrointestinal cancers, Health literacy, Quality of life, Patient activation, Oncology, Cancer, Psychology, Gastroenterology

## Abstract

Gastrointestinal (GI) cancers present significant health challenges, necessitating strategies to improve patients’ health-related quality of life (HRQoL). Health literacy (HL) and patient activation (PA) are key factors in patient self-management, yet their interplay and impact on HRQoL remain unclear. This study investigates the relationship between HL, PA, and HRQoL in GI cancer patients, with a focus on PA as a potential mediator between HL and HRQoL. We conducted a cross-sectional analysis using baseline data from the multicenter OptiScreen study. HL, PA, and HRQoL were assessed using validated instruments: the European Health Literacy Survey Questionnaire (HLS-EU-Q16), the Patient Activation Measure (PAM-13D), and the Short-Form Health Survey (SF-8). Statistical analyses included correlation tests and mediation modeling. Out of 854 eligible GI cancer patients, 397 (response rate = 46%) participated in the study. HL was positively correlated with PA, r(359) = 0.37, *p* < .001 and with physical and mental HRQoL, r(322) = 0.12, *p* = .035 and r(322) = 0.20, *p* < .001, respectively. We found that the relationship between HL and mental HRQoL is fully mediated by PA (indirect effect: 0.186, 95% CI [0.016, 0.385]). Our findings highlight the crucial role of PA in enhancing mental HRQoL in GI cancer patients, suggesting that interventions targeting both HL and PA could improve patient outcomes. Future research should explore phase-specific interventions and broader psychological factors affecting patient self-management and well-being.

## Introduction

The increasing global burden of GI cancers, with projected new cases reaching 7.5 million by 2040^1^, underscores the critical need for effective patient-centered care strategies^2^. As screening and treatment improve, the survivor population is growing, necessitating a focus on long-term health challenges faced by GI cancer patients^3^. Patients with GI cancers (gastric, liver, esophageal, pancreatic, and colorectal) are at a heightened risk of experiencing premature mortality due to the cancer itself, coexisting medical conditions, and exposure to cancer treatments^4^.

Cancers of the GI tract share several risk factors that are rooted in lifestyle factors such as unhealthy dietary habits^5^, physical inactivity^6^, obesity^7^, smoking, and alcohol consumption^8^. Mitigating these factors may enhance the tolerance of treatment and potentially improve survival rates^9,10^. A key component for empowering patients to make health-promoting decisions are self-management strategies, such as health literacy (HL)^11^ and patient activation (PA)^12^ which help patients adopt health-promoting behaviors to improve health outcomes^13–15^.

Health literacy is widely understood as a cognitive and social skill that plays a pivotal role in influencing individuals’ motivation and capacity to acquire, understand, and use information in manners that promote and maintain good health^16^. PA reflects knowledge, skills, confidence, and behaviors needed for self-managing one’s condition or health^16^. Both HL and PA are concepts enabling patients for self-managing their health. While PA frequently proves to be a more influential predictor of behaviors and health outcomes compared to HL, it is that HL can at times be a more robust predictor of comprehension and the utilization of information when making choices^17^. Hibbard et al. (2007) highlight that the ability to make informed choices, particularly when trade-offs are necessary, is associated with activation independently of comprehension. Individuals with higher activation are more likely to understand the consequences of their health-related decisions and prioritize quality in their choices. Higher activation can compensate for lower health literacy skills, especially in understanding health information and making quality decisions^18^.

While some studies have shown positive connections between HL and PA, these concepts show only a moderate correlation^19–21^. Research suggests further that PA and HL play distinct roles in influencing outcomes. Enhancing HL does not necessarily impact PA, and vice versa, so targeting both concepts may lead to more favorable health outcomes^19,20,22^.

To our knowledge, there are no studies that have examined the relationship of HL and PA on health-related quality of life in GI cancer patients. As this patient sample could especially benefit from a marked degree of HL and PA, we aim to assess these constructs in a homogenous sample of cancer patients. Our study is innovative in that it addresses both PA and HL, with a particular focus on the potential mediating role of PA. Building on previous findings that suggest PA can mitigate the effects of low HL, we examine how PA influences the impact of HL on Health-Related Quality of Life (HRQoL). Specifically, the primary aim of our research is to investigate the impact of HL on the mental and physical dimensions of HRQoL. The secondary aim is to examine patient activation, considering how PA may influence or moderate the effects of HL on HRQoL outcomes.

## Methods

### Study design and sample

The present study is based on cross-sectional data of the multicenter, non-randomized pre-post study “OptiScreen” aiming to evaluate the psychosocial screening procedure in cancer GI cancer patients^23^. A consecutive sample of inpatients was recruited at the respective visceral oncology ward at the three German Comprehensive Cancer Centers (Hannover, Leipzig, Dresden) at admission to the ward (baseline: t0) and after hospitalization (three months later: t1). We used the data of the first measuring point (t0). Patients were eligible for study participation if they had a (i) primary or recurrent diagnosis of a visceral cancer (ICD-10: C15-C26) were at (ii) age ≥ 18 years and had the (iii) cognitive ability to consent to study participation. Patients were excluded if they showed severe cognitive or physical impairments and language limitations. All participants gave written informed consent in accordance with the Declaration of Helsinki. The study was approved by the Research Ethics Committee from Hannover Medical School (8478_BO_K_2019), University Medical Center Leipzig (274/19-lk) and University Medical Center Dresden (EK 459,102,019).

### Study recruitment and data collection

Patient recruitment for t0 was carried out from 2020 June to 2021 September. Patients were approached by study staff when meeting the inclusion criteria and in consultation with the treating physicians/practitioners during their inpatient treatment on the respective visceral oncology ward. Study staff provided detailed information about the study. If patients agreed to participate in the study, a signed informed consent form was obtained, and participants were given a paper-pencil questionnaire to be completed during their inpatient stay (t0). Eligible patients who refused to participate were asked for a brief interview (3–5 min) to capture the reason for non-participation, whether they have received psycho-oncological care, assessment of current distress and to obtain sociodemographic and medical information on their GI cancer localization.

### Study measures

#### Sociodemographic and clinical data

Sociodemographic characteristics, i.e., sex, age, marital status, living with a partner, education, household income per month were obtained from patients’ self-reports. Clinical characteristics, i.e. cancer diagnosis, date of diagnosis, treatment indication, TNM classification, and received treatments were recorded based on patients’ medical charts and patients’ self-reports.

#### Health literacy

HL was assessed using the validated German version of the European Health Literacy Survey Questionnaire (HLS-EU-Q16)^24^. The HLS-EU-Q16 is clustered in four dimensions (‘access’, ‘understand’, ‘appraise’ and ‘apply’ health information) and three different domains (‘health care’, ‘disease prevention’, and ‘health promotion’). The 16 items are scored on a four-point Likert scale ranging from 1 ‘very difficult’ to 4 ‘very easy’ with higher scores indicating better health literacy^25^. The scale was dichotomized for scoring: very easy/ fairly easy = 1, fairly difficult/very difficult = 0. This score was summed and health literacy was categorized into three levels: ‘inadequate HL’: score 1–8, ‘problematic HL’: score 9–12, ‘adequate HL’: score 13–16^26^. Cases with more than two missing items were excluded^24,27^.

#### Patient activation

PA was assessed using the validated German version of the Patient Activation Measure (PAM-13D)^28^, a 13-item self-report measure assessing patient’s knowledge, skill and confidence for self-management of health conditions^11^. Respondents rate patient activation on a four-point Likert scale ranging from 1 ‘strongly disagree’ to 4 ‘agree strongly’. The sum score is ranging from 13 to 52 with higher scores representing higher levels of patient activation. Questionnaires with answers to seven or more items were included in the analyses. For better interpretability, the sum of the raw values was converted into natural logarithms and then to a standardized metric ranging from 0 to 100 (0 = lowest activation level, 100 = highest activation)^28^. The PAM-activation scores and levels were calculated by Insignia Health^29^.

#### Health related quality of life

We used the validated German version of the Short-Form Health Survey (SF-8) to measure general HRQoL^30^, a questionnaire which was originally a short-form health survey with 36 questions. The SF-8 comprises eight dimensions of HRQoL: general health (GH), physical functioning (PF), role physical (RP), bodily pain (BP), vitality (VT), social functioning (SF), mental health (MH), role emotional (RE). Items are rated on 5-point and 6-point scales. Physical (PCS) and mental component summary measures (PCS) were calculated by weighting each SF-8 item using a norm-based scoring method given in the instrument guidelines^30^. Higher summary PCS and MCS scores indicate better health.

### Statistical analysis

We applied descriptive analyses for both continuous (mean, standard deviation) and categorical variables (frequencies, percentages).

To examine differences between HL, HRQoL, and PA scores in socio-demographic and clinical characteristics t-tests or one-way analysis of variance (ANOVA) were calculated. Comparisons between participants and non-responders were made using ANOVA with Bonferroni correction due to multiple comparisons (adjusted α level 0.00625). Linear correlations between two variables were examined with bivariate correlation using Pearson’s r.

We computed two mediations using PROCESS model 4 with (1) PCS as outcome and (2) MCS as outcome representing HRQoL. Variables that were statistically significant (p < 0.05) in univariate analyses were included as covariates in both models. Mediating effects were estimated using linear regression models and interpreted as described by Baron and Kenny^31^ and MacKinnon^32^. First, following Baron and Kenny^31^, we assessed whether HL significantly predicts HRQoL by examining whether HL significantly influences PA in the initial regression equation (path a). Second, we examined whether HL significantly influences HRQoL (path c). Third, we evaluated whether PA mediates the relationship between HL and HRQoL by assessing the significance of paths b_1_ and b_2_ in the mediation models. If these conditions all hold in the predicted direction, then the effect of HL on HRQoL must be less in the third equation (path c’) than in the second (path c). Path c’ indicated the direct impact of HL on HRQoL after controlling for PA, which was considered partial mediation in case its regression coefficient was significant. According to MacKinnon’s guidance, the lack of statistical significance in path c does not necessarily preclude mediation. We further elaborate on the significance of paths a, b and c’. Bootstrap samples was set at 10.000 and level of confidence was set at 95.0.

Data analyses were performed with IBM SPSS Statistics 29^33^ and PROCESS Makro v4.2 for SPSS^34^.

## Results

### Sample

**Fig. 1:**
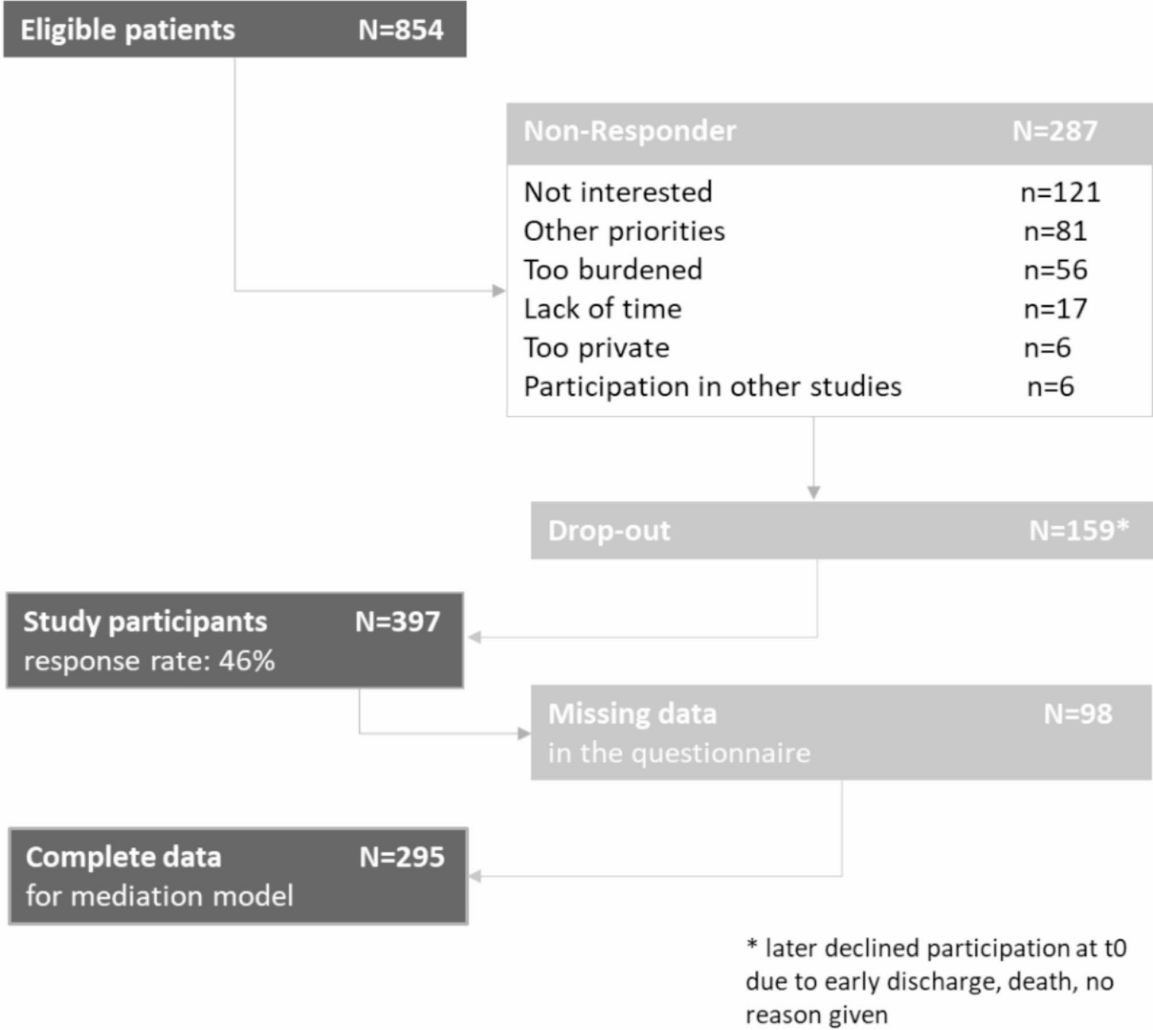
Flowchart of participants.

Out of 854 eligible patients, 397 (response rate = 46%) participated in the study (Fig. 1).

#### Non-responder analysis

Study participants were younger (M = 61 vs. M = 66 years, *p* < .001), differed in GI tumor location (*p* = .013) with a higher percentage of colon cancer (45.6% vs. 37.4%) and lower percentage of pancreas cancer (10.6% vs. 16.5%), and were more likely to receive curative cancer treatment (79.1% vs. 13%, *p* < .001) compared to non-responders. There were no significant differences in sex distribution among responders and non-responders.

#### Sample characteristics

Table 1 shows sociodemographic and clinical data for the total sample. The median age of participants was 61 years (IQR = 17). More than half of respondents were male (67.8%), married (71%) or living in a partnership (77.8%), having children (81.9%) and had a secondary educational degree or higher (81.4). Respondents were most commonly diagnosed with colon cancer (45.6%), had no metastases (50.1%), no second cancer diagnosis (80.1%), and were mostly treated with surgery (78.3%).

**Table 1:** Descriptive characteristics of the sample.

Characteristic	Number	Percentage
Total sample	397	100
Sociodemographic data		
Sex^a^		
Male	269	67.8
Female	121	30.5
Age in yrs^b^		
21–49	60	15.1
50–70	234	58.9
71–88	93	23.4
Marital status^c^		
Single	35	8.8
Married	296	74.6
Divorced	50	12.6
Living in separation	7	1.8
Widowed	23	5.8
Living in a joint household		
No	85	21.4
Yes	312	78.6
Having children		
No	72	18.1
Yes	325	81.9
Highest educational degree^d^		
Elementary educational degree (< 10 yrs)	63	15.9
Secondary educational degree (10 yrs)	173	43.6
High school degree (> 10 yrs)	150	37.8
Other	5	1.3
Household income in €/month^e^		
<1500	67	16.9
1500-<3000	169	42.6
≥3000	121	30.5
Clinical data		
Tumor location		
Anal	7	1.8
Bile duct	24	6.0
Stomach	42	10.6
Pancreas	42	10.6
Liver	47	11.8
Esophagus	54	13.6
Colon	181	45.6
Metastases^f^		
No	199	56.2
Yes	155	43.8
Second cancer diagnosis^g^		
No	318	80.1
Yes	78	19.6
Chemotherapy		
No	289	72.8
Yes	108	27.2
Radio-chemotherapy		
No	391	98.5
Yes	6	1.5
Radiotherapy		
No	382	96.2
Yes	15	3.8
Surgery		
No	86	21.7
Yes	311	78.3
Immune therapy		
No	387	97.5
Yes	10	2.5

yrs = years, ^a^N/A = 7, ^b^N/A = 10, ^c^multiple answers were possible, ^d^N/A = 6, ^e^N/A = 40, ^f^N/A = 43, ^g^N/A = 1.

### Scores of HL, PA and HRQoL

A total of 183 (46.1%) respondents met criteria for adequate HL, while 107 (27.0%) and 69 (17.4%) respondents met criteria for problematic and inadequate HL, respectively.

Mean scores and standard deviations for HL, PA, and HRQoL PCS and HRQoL MCS were 11.83 ± 3.45, 62.45 ± 15.27, 40.25 ± 11.47 and 46.19 ± 11.05, respectively. Table 2 presents the differences in mean scores for HL, PA, and HRQoL- PCS and MCS, categorized by sociodemographic and medical characteristics.

**Table 2:** Mean differences in PA, HL, and HRQoL categorized by sociodemographic and medical data.

	PA	HL	HRQoL	
			PCS	MCS
	M ± SD	M ± SD	M ± SD	
Sociodemographic data				
Sex^a^				
Male	60.17 ± 14.63	11.87 ± 3.47	40.86 ± 11.59	46.85 ± 10.51
Female	63.70 ± 15.95	11.88 ± 3.38	38.63 ± 11.22	44.72 ± 12.06
*t*	-2.141	-0.020	1.634	1.624
*p*	0.033*	0.984	0.103	0.105
Age in yrs^b^				
21–49	60.89 ± 14.89	12.10 ± 3.00	41.07 ± 12.60	44.60 ± 11.03
50–70	60.96 ± 15.02	11.84 ± 3.60	39.98 ± 11.14	46.27 ± 10.40
71–88	62.81 ± 15.94	11.78 ± 3.33	40.66 ± 11.82	46.77 ± 12.32
*F*	0.529	0.166	0.236	0.673
*p*	0.590	0.847	0.790	0.511
Marital status^c^				
Single	54.34 ± 12.65	11.38 ± 3.39	37.69 ± 13.14	45.26 ± 10.31
Married	62.23 ± 14.42	11.93 ± 3.41	40.67 ± 11.40	46.04 ± 10.92
Divorced	61.75 ± 18.39	11.60 ± 3.57	39.56 ± 11.43	46.29 ± 13.11
Living in separation	62.11 ± 19.05	13.83 ± 2.40	50.47 ± 8.49	51.16 ± 9.03
Widowed	60.32 ± 16.73	11.67 ± 4.04	36.48 ± 10.01	48.10 ± 9.95
*F*	1.951	0.735	1.654	0.369
*p*	0.101	0.568	0.160	0.831
Living in a joint household				
No	59.90 ± 16.71	11.45 ± 3.49	39.92 ± 11.34	45.20 ± 11.70
Yes	61.87 ± 14.83	11.93 ± 3.44	40.34 ± 11.53	46.46 ± 10.87
*t*	-1.065	-1.069	-0.273	-0.865
*p*	0.287	0.286	0.785	0.388
Having children				
No	55.85 ± 14.40	11.65 ± 3.52	40.05 ± 11.66	44.26 ± 10.10
Yes	62.67 ± 15.20	11.87 ± 3.44	40.29 ± 11.45	46.61 ± 11.22
*t*	-3.479	-0.467	-0.152	-1.512
*p*	< 0.001***	0.641	0.879	0.132
Highest educational degree^d^				
Elementary educational degree (< 10 yrs)	62.51 ± 16.20	12.92 ± 3.28	41.67 ± 11.72	48.72 ± 10.06
Secondary educational degree (10 yrs)	60.54 ± 15.40	11.22 ± 3.70	38.33 ± 11.79	45.87 ± 11.29
High school degree (> 10 yrs)	61.66 ± 14.34	12.13 ± 3.01	41.95 ± 10.81	45.94 ± 10.89
Other	77.46 ± 25.00	11.75 ± 5.31	44.02 ± 4.49	37.68 ± 15.82
*F*	2.143	3.900	2.859	1.718
*p*	0.094	0.009**	0.037*	0.163
Household income in €/month^e^				
<1500	61.16 ± 18.02	11.49 ± 3.36	37.14 ± 12.30	45.11 ± 12.39
1500-<3000	61.08 ± 15.29	11.62 ± 3.59	40.07 ± 11.33	45.85 ± 11.10
≥3000	60.88 ± 13.27	12.06 ± 3.34	42.21 ± 11.07	46.66 ± 10.21
*F*	0.009	0.734	3.543	0.371
*p*	0.991	0.481	0.030*	0.690
Clinical data				
Tumor location				
Anal	66.57 ± 23.96	10.43 ± 5.68	41.62 ± 14.13	47.50 ± 10.23
Bile duct	64.17 ± 16.19	11.95 ± 2.95	41.17 ± 12.53	45.71 ± 9.77
Stomach	58.39 ± 12.98	11.97 ± 3.03	39.19 ± 12.30	46.38 ± 10.77
Pancreas	62.01 ± 15.23	11.34 ± 3.87	35.59 ± 11.54	38.54 ± 13.74
Liver	63.38 ± 15.61	11.79 ± 3.65	41.85 ± 9.49	50.33 ± 9.50
Esophagus	60.97 ± 17.09	11.29 ± 3.53	39.59 ± 10.98	45.36 ± 11.41
Colon	61.08 ± 14.67	12.11 ± 3.34	41.11 ± 11.51	46.87 ± 10.44
*F*	0.698	0.707	1.323	3.178
*p*	0.651	0.645	0.246	0.005**
Metastases^f^				
No	60.12 ± 14.97	11.53 ± 3.49	39.78 ± 11.51	46.01 ± 11.24
Yes	62.80 ± 15.76	12.11 ± 3.31	40.41 ± 11.65	46.16 ± 11.21
*t*	-1.624	-1.518	0.478	0.110
*p*	0.105	0.130	0.633	0.912
Second cancer diagnosis^g^				
No	63.58 ± 15.57	12.05 ± 3.47	40.07 ± 11.53	46.03 ± 10.64
Yes	60.89 ± 15.19	11.78 ± 3.45	40.70 ± 11.17	46.69 ± 12.68
*t*	1.366	0.568	0.399	0.393
*p*	0.173	0.570	0.464	0.695
Chemotherapy				
No	61.43 ± 15.40	12.00 ± 3.41	41.27 ± 11.20	46.61 ± 11.15
Yes	61.45 ± 14.98	11.39 ± 3.53	37.52 ± 11.80	45.08 ± 10.76
*t*	-0.017	1.518	2.716	1.136
*p*	0.986	0.130	0.007**	0.250
Radio-chemotherapy				
No	61.45 ± 15.35	11.80 ± 3.44	40.36 ± 11.45	46.24 ± 11.10
Yes	60.18 ± 8.83	13.33 ± 3.88	34.08 ± 12.25	43.45 ± 7.91
*t*	0.203	-1.078	1.329	0.612
*p*	0.840	0.282	0.185	0.541
Radiotherapy				
No	61.50 ± 15.20	11.89 ± 3.43	40.35 ± 11.47	46.35 ± 11.00
Yes	59.67 ± 17.45	10.36 ± 3.73	37.32 ± 11.71	41.78 ± 12.02
*t*	0.456	1.629	0.900	1.410
*p*	0.649	0.104	0.369	0.160
Surgery				
No	58.76 ± 14.99	11.76 ± 3.12	37.50 ± 11.12	46.30 ± 11.79
Yes	62.17 ± 15.28	11.84 ± 3.53	40.94 ± 11.48	46.17 ± 10.87
*t*	-1.840	-0.189	-2.236	0.087
*p*	0.067	0.850	0.026*	0.930
Immune therapy				
No	61.45 ± 15.23	11.83 ± 3.44	40.22 ± 11.51	46.25 ± 11.11
Yes	60.80 ± 17.33	11.56 ± 3.94	41.51 ± 10.52	43.95 ± 8.48
*t*	0.134	0.239	-0.238	0.617
*p*	0.894	0.811	0.812	.

PA = patient activation measured with PAM; HL = health literacy measured with HLS, HRQoL = health related quality of life measured with SF-8, PCS = physical component scale, MCS = mental component scale (subscales of SF-8), M = mean, SD = standard deviation, F = F-statistic, t = t-statistic, yrs = years, p = level of statistical significance based on F-tests and t-tests, ^a^N/A = 7, ^b^N/A = 10, ^c^N/A = 4, ^d^N/A = 6, ^e^N/A = 40, ^f^N/A = 43, ^g^N/A = 1, ^*^significant on a level of *p* < .05, ^**^significant on a level of *p* < .01, ^***^significant on a level of *p* < .001.

### The relationship between HL, PA, and HRQoL

HL scores were significantly correlated with PA scores, r(359) = 0.37, *p* < .001, where 359 indicates the degrees of freedom. Additionally, HL scores showed significant correlations with both HRQoL PCS, r(322) = 0.12, *p* = .035 and HRQoL MCS, r(322) = 0.20, *p* < .001. PA scores were significantly correlated with both HRQoL PCS and HRQoL MCS scores, r(342) = 0.17, *p* = .001 and r(342) = 0.17, *p* = .002.

### Mediation role of PA on HL and HRQoL

**Fig. 2:**
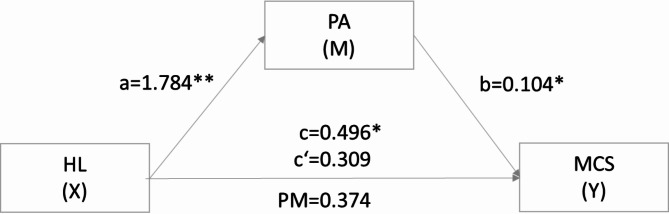
Mediation model of the indirect effects of health literacy on the mental component of health related quality of life. c = total effect of X on Y; c’ = direct effect of X on Y through M or a*b. HL = health literacy, PA = patient activation, MCS = mental component scale, PM = proportion mediated, ratio of direct to indirect effect.

Table 3 shows the mediating effects of PA with the summarized coefficients and significance values found in the two mediation models. After controlling for all sociodemographic and clinical data (i.e., sex, children, highest educational degree, household income, tumor location, chemotherapy, and surgery) that were found to be significant in the previous analyses, the first mediation analysis revealed that HL had a significant total effect on MCS (path c: B = 0.496, *p* = .014). After entering the mediator in the model, HL predicted the mediator PA significantly (path a: B = 1.784, *p* < .001), which in turn predicted MCS significantly (path b: B = 0.104, *p* = .039). HL had no significant effect on MCS after controlling for PA (path c’: B = 0.309, *p* = .155). The relationship between HL and MCS is fully mediated by PA, indirect effect ab = 0.186, 95%-CI[0.016, 0.385] (Fig. 2).

**Fig. 3:**
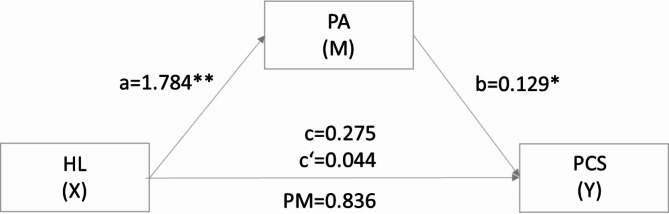
Mediation model of the indirect effects of health literacy on the physical component of health related quality of life. c = total effect of X on Y; c’ = direct effect of X on Y through M or a*b. HL = health literacy, PA = patient activation, PCS = physical component scale, PM = proportion mediated, ratio of direct to indirect effect.

The second mediation analysis revealed that HL had no significant total effect on PCS (path c: B = 0.275, *p* = .147). After entering the mediator in the model, HL predicted the mediator PA significantly (path a: B = 1.784, *p* < .001), which in turn predicted PCS significantly (path b: B = 0.129, *p* = .020). HL had no significant effect on PCS after controlling for PA (path c’: B = 0.044, *p* = .836). There was no mediating effect of PA on HL and PCS (Fig. 3).

**Table 3:** Summary of the mediating effects of PAM on the relationship between HL and MCS (*n* = 295) and PCS (*n* = 295).

Type	Effect	*B*	*SE*	t	*p*	95% CI^a^	
						Lower	Upper
Indirect							
	HL→PA→MCS	0.186	0.094	-	-	0.016	0.385
	HL→PA→PCS	0.230	0.103	-	-	0.041	0.443
Component							
	HL→PA	1.784	0.244	7.302	< 0.001	1.303	2.265
	PA→MCS	0.104	0.050	2.074	0.039	0.005	0.203
	PA→PCS	0.129	0.055	2.330	0.020	0.020	0.238
Direct effects							
	HL→MCS	0.309	0.217	1.423	0.155	-0.118	0.738
	HL→PCS	0.044	0.215	0.206	0.836	0.380	0.469
Total effects							
	HL→MCS	0.496	0.202	2.454	0.014	0.098	0.894
	HL→PCS	0.275	0.189	1.452	0.147	-0.097	0.647

^a^number of bootstrap samples for percentile bootstrap confidence intervals: 10,000.

*B* = standardized coefficient, *SE* = standard error, CI = confidence interval, *t* = *t*-value, *p* = *p*-value, HL = health literacy, PA = patient activation, PCS = physical component scale, MCS = mental component scale.

## Discussion

In this study, we examined associations between HL, PA and HRQoL, and we were able to demonstrate a mediation effect of HL and PA on the mental domain of HRQoL. Nearly half of the respondents in our sample showed problematic or inadequate HL. However, our sample demonstrated higher HL compared to adolescents and young adult (AYA) cancer patients and the norm population in Germany (28 and 46% with adequate HL)^26,35^. Surprisingly, in our study, participants with less than 10 years of schooling had significantly higher HL compared to those with higher education. This is in contrast with several previous findings showing higher HL in respondents with higher educational level^36–38^. However, HL and the educational level are not identical concepts and the relationship is not fully understood^39^. Insufficient HL was also found in individuals with a higher educational level, and HL does not increase linearly with educational level^38^. In addition, it is well known that self-report questionnaires do not necessarily reflect the true level of an individual’s competencies. This observation is known as the Kruger-Dunning effect, wherein individuals tend to overestimate their competence in logical reasoning and knowledge, particularly those who perform poorly^40^. A study investigating HL and health behavior in a community sample of *N* = 504 participants confirmed this effect. It disclosed that individuals with low HL not only expressed comparable or higher confidence in their health knowledge compared to those with higher HL but also displayed more problematic health behaviors, such as smoking^41^.

In our sample, overall PA was high. Individuals having children and female respondents showed higher PA scores. There are mixed results regarding a sex effect on PA and HL in the literature. While higher PA was found in breast cancer survivors compared to prostate cancer survivors^42^, it was also shown that both men and women experience similar significant limitations in HL in a range of national and international studies^43^.

Overall, respondents showed better mental HRQoL compared to physical HRQoL in our study. We found significantly higher scores in PCS for individuals not receiving surgery and undergoing chemotherapy. MCS scores were significantly higher in individuals undergoing chemotherapy. It seems counterintuitive that respondents undergoing chemotherapy have a higher HRQoL than those without chemotherapy. However, the majority of our respondents received surgery instead of chemotherapy. Surgery can be associated with long recovery periods with severe physical limitations, which could exceed the side effects of other therapies like chemotherapy, therefore leading to worse HRQoL^44^.

In our sample, HL was significantly positively correlated with PA and HRQoL, i.e. lower HL is associated with lower PA and HRQoL. Overall, correlations were small to moderate. This is consistent with findings of a study examining older people with long-term conditions, showing that PA was significantly lower in patients with poor HL^45^. Here, another important associated factor with low PA was impaired HRQoL. According to the results of our correlation analyses, HL and PA appear to measure different constructs, but show some overlap. Therefore, it is important to consider both HL and PA, as suggested in the literature^17,19^.

We found that the relationship between HL and mental HRQoL is fully mediated by PA, whereas we found no such effect for physical HRQoL. This is in line with results of a study by Vohra et al. examining individuals with pancreatic cancer. Here, the authors found significant positive associations between PA and emotional HRQoL, but no associations between PA and physical HRQoL^15^. As many patients in our study were in a recovery process after long surgical procedures at the time of examination, the patient’s capacity to improve the own physical condition may be limited. Thus, physical recovery at that stage may not be accelerated by higher HL or PA.

### Study strengths and limitations

Due to Covid-19 restrictions, study personnel were temporarily prohibited from accessing patients for enrollment, resulting in a smaller sample size than planned. Our response rate was relatively low because study participants were burdened due to various reasons, such as inpatient treatment at the time of data collection and therefore may have been more likely to refuse to participate in this study. Since the collected data was self-reported, and respondents were asked about behavior on health-related questions, data is prone to response biases like social desirability or recall bias. Finally, owing to the cross-sectional nature of the data utilized, causal relationships cannot be established.

A strength of our study is the consideration of both HL and PA, and the results contribute to the knowledge of the complex relationship between HL, PA and HRQoL. Our sample consists of inpatients with GI tumors; therefore, our findings may be more representative of individuals treated in clinical sites. However, the generalizability of our results to other tumor entities should not be necessarily limited, as one’s health-related knowledge and behavior probably does not differ between cancer types.

### Implications for further research and clinical applications

Our study underscores the significant roles of HL and PA in influencing the mental aspect of HRQoL among GI cancer patients. Enhancing HL and PA through targeted interventions has the potential to improve mental well-being and overall HRQoL in this patient population. Such interventions should prioritize patient education and empowerment by delivering comprehensive information regarding the disease, available treatment options, and self-care strategies^46^. This approach might enhance patients’ confidence and motivation to participate in various activities, including those related to physical activity, medication adherence, dietary management, and stress reduction. Equipping patients with self-management skills, such as problem-solving, decision-making, and coping strategies enhances their ability to navigate challenges effectively, thereby improving psychological well-being. Finally, continuous support and monitoring of patient progress, with regular feedback and adjustments to care plans, are crucial for sustaining motivation and promoting active patient engagement in self-management practices^47,48^.

As HRQoL is a broad term for a person’s well-being and level of functioning, it could be useful in a next step to examine associations between HL, PA and typical psychological distress in cancer (e.g. fear of recurrence, depression). A study by Magnezi et al. (2014) showed that lower PA was associated with higher levels of depressive symptoms and lower quality of life in patients with chronic conditions. The authors concluded that active participation in one’s own health management could be an expression of psychological well-being^49^. Thus, surveying PA and psychological conditions could provide an indication of the extent to which patients are able to comply with health recommendations in the first place. Strategies that promote higher levels of PA may mitigate depressive symptoms and improve overall quality of life. Such strategies may include the integration of mental health services for recognizing and addressing the psychological aspects of chronic conditions through integrated care models that ensures holistic support for patients.

A large nationwide study found that HL in the German population is overall low, but particularly poor in vulnerable groups, such as individuals with chronic conditions^50^. In their study, particular challenges arose in understanding health relevant information as well as the practical application of health information, especially in the area of illness management. Policy makers should consider initiatives to enhance HL among vulnerable groups, potentially through educational programs and improved accessibility of health information. This can be achieved by addressing health literacy barriers by using plain language, visual aids, and culturally appropriate materials^51^. Providing understandable information and clear instructions on health behavior could make it easier for patients to take a more active role in their own health management, thereby improving mental wellbeing as well.

As many participants in our study were in recovery post-surgery, further investigation into the mediation effect between HL, PA, and physical HRQoL in different phases of disease management is warranted. Tailoring interventions based on disease phases could optimize health outcomes across the spectrum of cancer care.

Our study suggests that integrating HL and PA in interventions tailored to cancer patients could improve mental well-being and overall HRQoL. Addressing psychological distress and advocating for policies that enhance HL are crucial implications for clinical practice and future research.

## Data Availability

The datasets generated during and/or analysed during the current study are available from the corresponding author on reasonable request.
